# The emergence of targetable MEKanisms in sporadic lymphatic disorders

**DOI:** 10.15252/emmm.202012822

**Published:** 2020-09-18

**Authors:** Michael T Dellinger, Francis X McCormack

**Affiliations:** ^1^ Division of Surgical Oncology Department of Surgery and the Hamon Center for Therapeutic Oncology Research UT Southwestern Medical Center Dallas TX USA; ^2^ Division of Pulmonary, Critical Care, and Sleep Medicine University of Cincinnati Medical Center Cincinnati OH USA

**Keywords:** Vascular Biology & Angiogenesis

## Abstract

Sporadic lymphatic diseases are orphans among orphans in the medical community, a diverse collection of disorders at the intersection of cardiac, gastrointestinal, pulmonary, dermatologic, and oncologic disease that receives only passing attention in medical school and that no subspecialty in medicine fully embraces as its own. They often present in a confusing and illusive manner, with a fractured bone, expectoration of blood or a branching airway cast, a swollen limb or a collection of chylous material; protean manifestations that can challenge even the most expert diagnostician. Yet many of these acquired disorders have been discovered to have a targetable genetic basis, and as the case report of Foster *et al* (2020) demonstrates, the sedulous clinician–patient dyad can be rewarded with an almost miraculous result when the molecular pathogenesis of the disease is pursued and an exquisitely targeted therapy is administered.

## Graphical abstract

F. McCormack and M. Dellinger highlight an astonishing result in treatment of Kaposiform lymphangiomatosis presented by J. Foster and colleagues, in this issue of *EMBO Molecular Medicine*.

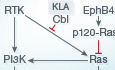

Each day, the lymphatic system resorbs 1–2 liters of protein‐rich fluid from the interstitium of vascularized tissues and returns it to the venous system via a one‐way vascular tree that originates with blind‐ended, single‐cell thickness vessels that connect to larger conducting vessels and ultimately to the thoracic duct and subclavian vein (Tammela & Alitalo, 2010; Fig 1A). Dietary fats resorbed by lacteals of the small intestine are discharged into the flow, which also serves as a conduit for leukocytes that are extravasated from the blood circulation and recovered by lymphatic capillaries to interact with networks of lymph nodes and ultimately returned to the bloodstream armed with vital targeting directives. Intrinsic contractile action of the lymphatic conducting vessels and muscular action in the extremities propels fluid caudad, with retrograde flow prevented by intravascular valves. The lung lymphatics are a parallel system with lymphatic flow that is primarily propelled by the bellows action of respiration and that drains into the thoracic duct.

**Figure 1:**
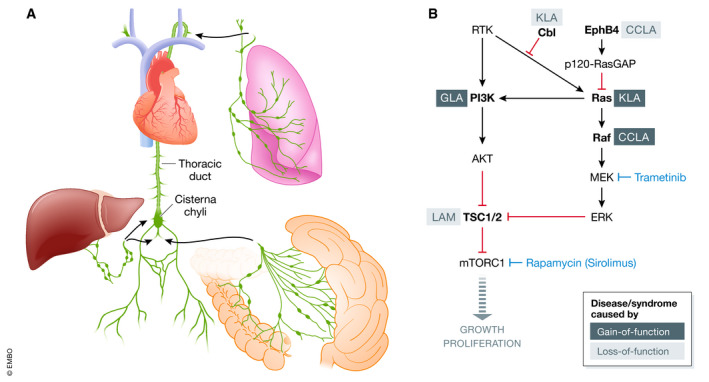
Lymphatic anatomy, genetic basis of sporadic lymphatic disorders, and targetable downstream signaling nodes (A) The lymphatic channels from the lower extremities, intestines, and liver converge on the cisterna chyli and drain into the subclavian vein via the thoracic duct. Genetic mutations that cause dysplasia or obstruction of lymphatic vessels can result in chylous ascites, chylothorax, chyluria, protein‐losing enteropathy, or plastic bronchitis. Embolization via the thoracic duct can be attempted for downstream leaks, but is more difficult when addressing leaks from countercurrent, feeding circuits and tributaries that arise from the liver and mesentery. (B) Gain‐of‐function and loss‐of‐function mutations in the PI3K/Akt/mTOR and MAPK pathways can lead to sporadic lymphatic disorders, many of which are amenable to targeted therapies approved for other indications, such as sirolimus and trametinib. CCLA, central conducting lymphatic anomaly; GLA, generalized lymphatic anomaly; KLA, kaposiform lymphangiomatosis; LAM, lymphangioleiomyomatosis.

Sporadic lymphatic diseases that interfere with this vascular system can result in disruption of fluid homeostasis, nutrition, and immune function (Fig 1B). These include lymphangioleiomyomatosis (LAM) (Henske & McCormack, 2012), yellow nail syndrome (YNS), and those collectively termed the complex lymphatic anomalies (CLAs), including generalized lymphatic anomaly (GLA), Gorham–Stout disease (GSD), central conducting lymphatic anomaly (CCLA), and kaposiform lymphangiomatosis (KLA) (Trenor & Chaudry, 2014). Patients with these diseases can have tortuous dilated lymphatics, diffuse multifocal lymphatic malformations, dilated lacteals with protein‐losing enteropathy, and ectopic lymphatics in bone and osteolytic lesions (Trenor & Chaudry, 2014). Lymphedema, pleural effusions, and ascites occur when antegrade lymphatic flow is obstructed or impeded by extrinsic compression from lymphatic masses, intraluminal obstruction, dysplastic transformation, or valvular destruction. Patients with disease above the level of the cisterna chyli can also present with chylous fluid accumulations, leakages or discharges, protein‐losing enteropathy or plastic bronchitis that results from reflux of lymphatic fluids containing chylomicrons into the pulmonary lymphatics, potential spaces, hollow viscera, airways, or fistulous tracks. Recent advances in lymphatic imaging including T2‐weighted MRI and MR lymphangiography have largely replaced lymphoscintigraphy and can reveal ectopic or dysplastic lymphatic masses, lymphatic leaks, and sites of lymphatic obstruction (Itkin & McCormack, 2016). Although new approaches to thoracic duct cannulation and embolization can be curative for refractory chylous effusions, plastic bronchitis, and chylous ascites, these techniques do not impact underlying disease pathogenesis or the effect of lymphatic disease on fluid homeostasis, pulmonary function, or osseous integrity.

Somatic mutations in *TSC2* (Henske & McCormack, 2012), *PIK3CA* (Rodriguez‐Laguna *et al*, 2019), *ARAF* (Li *et al*, 2019), and *NRAS* (Barclay *et al*, 2019) have been found in LAM, GLA, CCLA, and KLA patients, respectively (Fig 1B). Interestingly, these same mutations occur in cancer and cause either inappropriate PI3K/AKT/mTOR or MAPK signaling (Trenor & Chaudry, 2014). Sirolimus and everolimus are mTOR inhibitors that act downstream of most lymphatic disease driving mutations in the PI3K/AKT/mTOR pathway and have been proven to be remarkably effective at stabilizing or reversing disease manifestations in some patients with LAM, GLA, CCLA, and KLA (McCormack *et al*, 2011; Adams *et al*, 2016). However, not all adult lymphatic disease patients improve with sirolimus/everolimus (Adams *et al*, 2016) and it is clear we have much more to learn about the genetic basis and dysregulated signaling underlying these disorders.

In this issue of *EMBO Molecular Medicine*, Foster *et al* (2020) describe a novel mutation and treatment for kaposiform lymphangiomatosis (KLA) in a patient with pericardial and pleural effusions, and who also had involvement of her mediastinum, lungs, right breast, axilla, spleen, and several bones. Although her disease had remained stable for many years on sirolimus, it worsened after discontinuing the drug and became unresponsive to sirolimus on a subsequent trial. Genetic analysis of endothelial cells isolated from her pleural fluid revealed that she had a somatic loss‐of‐function mutation in *CBL* (c.2322T>G; p.Y774*), a E3 ubiquitin–protein ligase that negatively regulates the RAS/MAPK pathway. Because the patient's mutation was predicted to increase RAS/MAPK signaling, she was treated with trametinib, an FDA‐approved MEK inhibitor. The patient's disease rapidly improved following treatment and she experienced a near‐complete resolution of her symptoms. Interestingly, lymphatic imaging revealed a remarkable “remodeling” of the drainage pattern of her lymphatic system after treatment.

The exciting findings by Foster *et al* (2020) shed new light on the pathogenesis of KLA, including insights into the genetics and pharmacotherapeutic approaches to the disease. Many unanswered questions still exist, including the mechanism by which CBL regulates RAS/MAPK signaling in lymphatic endothelial cells and leads to destructive remodeling, and whether there are differences in phenotype, prognosis, circulating angiopoietin‐2 levels [a diagnostic biomarker for KLA (Le Cras *et al*, 2017)], or response to treatment between KLA patients with a CBL or NRAS mutation. What is clear is that supportive care alone is no longer the standard of care for patients with lymphatic disease. Clinicians who encounter these diseases are well advised to search for the genetic basis and deliver targeted therapies when available or refer to a specialized center with the expertise and resources to pursue a molecular diagnosis.

In conclusion, the paper by Foster *et al* (2020) offers new hope for patients suffering from KLA and all lymphatic disorders. Importantly, next‐generation sequencing studies like the one described in this issue of *EMBO Molecular Medicine* could lead to the repurposing of additional FDA‐approved pharmacotherapies for KLA and other CLAs and usher in a new era of precision medicine for the treatment of these life‐threatening diseases.
